# 4165 Mechanisms of Prophage-Mediated Virulence Driving Community-Acquired MRSA Contagion

**DOI:** 10.1017/cts.2020.79

**Published:** 2020-07-29

**Authors:** Robert James Ulrich, Irnov Irnov, William Sause, Magdelena Podkowik, Victor Torres, Bo Shopsin

**Affiliations:** 1 NYU - H+H Clinical and Translational Science Institute; 2 Stanford University; 3 NYU School of Medicine

## Abstract

OBJECTIVES/GOALS: We recently identified a CA-MRSA strain in Brooklyn, New York (USA300-BKV) causing an outbreak of severe skin infections in predominantly healthy children. The evolution of USA300-BKV included acquisition of a novel prophage, and our objective is to identify the prophage-encoded gene(s) and mechanism responsible for increased bacterial virulence. METHODS/STUDY POPULATION: We deleted candidate genes from a novel mosaic block of phage-encoded genes in USA300-BKV that have been shown to enhance virulence in a murine skin infection model. Deletion mutants and complemented clones will be evaluated *in vivo* to identify culprit genes and determine the effect of lineage-specific genetic variation on the phenotype. Complementary studies include a comprehensive characterization of phage and bacterial genes expressed during lysogeny *in vitro* using RNA sequencing (RNA-Seq), and *in vivo* using a targeted approach focusing on known bacterial virulence and phage lytic pathways as well as candidate genes identified by *in vitro* studies. RESULTS/ANTICIPATED RESULTS: Comparison of otherwise isogenic lab strains showed that the mosaic block of phage genes present in USA300-BKV enhance skin abscess size in mice, confirming previous results. As this region of the phage, named mΦ11, does not contain known toxin genes, we hypothesize that mΦ11 modulates expression of bacterial host genes to enhance virulence. Thus, transcriptional profiles of CA-MRSA containing mΦ11 and selected deletion mutants are expected to reveal changes in known or novel virulence factors compared to controls. Candidate regulators specific to the mosaic block include an adenine methyltransferase linked to changes in global gene expression of other bacterial species. DISCUSSION/SIGNIFICANCE OF IMPACT: Our results will broaden scientific understanding of phage-bacterial interactions and determine the mechanisms by which phage impact virulence independent from toxin gene carriage. Identification of phage-encoded gene(s) enhancing CA-MRSA contagion will inform surveillance efforts and identify novel therapeutic targets.

